# Inconsistent condom use and its associated factors among female sex workers in African countries: Systematic review and meta-analysis

**DOI:** 10.1371/journal.pone.0346903

**Published:** 2026-04-10

**Authors:** Mulat Belay Simegn, Habtamu Geremew, Fraol Daba Chinkey, Zekarias Tadele Alemneh, Eyasu Bamlaku Golla, Alegntaw Abate, Mohammed Ahmed Ali, Smegnew Gichew Wondie, Hawi Kumbi, Werkneh Melkie Tilahun

**Affiliations:** 1 Department of Public Health, College of Medicine and Health Sciences, Debre Markos University, Debre Markos, Ethiopia; 2 College of Health Science, Oda Bultum University, Chiro, Ethiopia; 3 Department of Internal Medicine, College of Medicine and Health Science, Ambo University, Ambo, Ethiopia; 4 Department of Internal Medicine, College of Medicine and Health Science, Aksum University, Aksum, Ethiopia; 5 Department of Medical Laboratory Science, College of Health Science, Oda Bultum University, Chiro, Ethiopia; 6 Department of Midwifery, College of Health Science, Oda Bultum University, Chiro, Ethiopia; 7 Department of Human Nutrition, College of Medicine and Health Science, Mizan Tepi University, Mizan Aman, Ethiopia; 8 Department of Laboratory, Adama Hospital Medical College, Adama, Ethiopia; Johns Hopkins Bloomberg School of Public Health, USAID/Public Health Institute, UNITED STATES OF AMERICA

## Abstract

**Background:**

Inconsistent condom use represents the most proximal behavioral risk factor for acquisition and transmission of sexually transmitted infections, including human immunodeficiency virus. However, certain situations hinder female sex workers from practicing consistent condom use. This study aimed to assess the pooled estimate of inconsistent condom use among female sex workers and identify factors associated with it.

**Methods:**

This study was conducted in accordance with the Preferred Reporting Items for Systematic Reviews and Meta-Analyses, 2020 reporting checklist. Electronic databases (PubMed, Cochrane Library, Epistemonikos, Hinari, and Science Direct), Google Scholar, and other university repositories were searched until March 20, 2024, based on the eligibility criteria. Three independent reviewers screened the titles, abstracts, and full texts. Two independent reviewers extracted the data. The Joanna Briggs Institute quality appraisal checklist was used. The Higgin’s I² test was used to quantify heterogeneity. Pooled analysis was conducted using a random-effects model. Sensitivity and subgroup analyses were done. Publication bias was assessed using Egger’s regression test and funnel plot. The pooled prevalence and statistical association were declared at a p-value < 0.05 with the 95% CI.

**Results:**

A total of 24 studies involving 23,496 female sex workers with a median age of 27.3 years were included. The overall pooled prevalence of inconsistent condom use among FSW in Africa was estimated at 46.73% (95% CI: 37.60, 55.86), I² = 99.59%, and p = 0.00. Condom availability (AOR = 0.68; 95% CI: 0.50, 0.92), depression (AOR = 1.51; 95% CI: 1.00, 2.30), no education (AOR = 1.87; 95% CI: 1.19, 2.93), two or more nonpaying clients (AOR = 2.90; 95% CI: 1.51, 5.54), having >9 current client number (AOR = 0.46; 95% CI: 0.29, 0.74), violence (AOR = 1.74; 95% CI: 1.33, 2.27), and police harassment (AOR = 2.28; 95% CI: 1.03, 5.05) were significant factors.

**Conclusion and recommendation:**

Inconsistent condom use was high in Africa. Factors including availability of condoms, depression, and education, having two or more nonpaying clients, client numbers, violence, and police harassment were significant factors. Strategies like improving peer education, providing mental health support, empowering women, and improving female sex workers educational status, ensuring condom availability, and strengthening supply for easily accessible condoms can decrease inconsistent condom use and protect FSWs from STI including HIV.

## Introduction

Human immunodeficiency virus (HIV) infection remains a global challenge that affects about 38 million individuals, and nearly 70% of them are living in sub-Saharan African countries [1]. Female sex workers (FSW) in African countries are disproportionately affected by HIV. For instance, a study reported that 19% of new HIV infections in Western and Central Africa occur among sex workers, the highest proportion across all regions [2]. In Ethiopia, the HIV prevalence among FSWs is estimated to be around 18.5% [3]. One important risk group for HIV is FSWs, whose risk is 14 times higher than that of women in general population [4].

Inconsistent condom use (ICU) among FSWs in African countries remains a significant public health concern, contributing to the elevated risk of HIV [5]. Recent studies have highlighted varying prevalence rates of inconsistent condom use among FSWs across different regions. For instance, in Ethiopia, ICU is prevalent, and factors like educational status, alcohol use, depression, and violence were identified [3]. Similarly, in Uganda, it was indicated that 33.3% to 55.1% of FSWs reported inconsistent condom use [6]. Promotion of condoms remains central to all the HIV/AIDS intervention strategies [7]. When used correctly and consistently, condoms are a very affordable and effective method of preventing the spread of sexually transmitted illnesses, including HIV, genital warts, cervical cancer, and other disorders linked to the human papillomavirus [8]. Review studies have shown that behavioral interventions have improved the use of condoms [9]. However, behavioral and structural issues frequently provide obstacles for female sex workers, who are most in need of using condoms with their relationships [10]. Research has suggested that these obstacles are all related to the stigma, discrimination, disempowerment, and lack of community involvement experienced by these marginalized groups [11]. The challenges tend to vary according to the context of sex work, countries, and typology of workers [12,13]. Inconsistent condom use represents the most proximal behavioral risk factor for HIV acquisition and onward transmission among female sex workers. Inconsistent condom use is defined as not using condom always during sexual encounter within a specified recall period. However, this definition may not be consistent across different studies, which may use different time periods (last sex act, last one week, and last one month etc.) and leading to discrepancies in the prevalence estimate. Despite the high risk of inconsistent condom use among FSW, there is still a dearth of summarized information in Africa. Understanding the prevalence and associated risk factors of ICU among FSW in Africa is essential for developing targeted intervention strategies and informing policymakers to promote safer sexual practices, thereby reducing HIV transmission within this vulnerable population.

## Methods and materials

This study was conducted in accordance with the Preferred Reporting Items for Systematic Reviews and Meta-Analyses, 2020 (PRISMA-2020) reporting checklist (**S2 File**) [14]. The protocol for this study was submitted and registered at PROSPERO with a registration number of CRD42024520159. The databases, including PubMed, Epistemonikos, PROSPERO, and the Cochrane Library were searched for similar systematic reviews to avoid duplications, and we found that it had not been conducted previously.

### Review question

1. What is the pooled prevalence of ICU among female sex workers in Africa? and
2. What are the factors associated with ICU among female sex workers in Africa?

### Measurement of Outcomes

The first outcome of this review and meta-analysis was the prevalence of inconsistent condom use among FSW. Inconsistent condom use was defined as the failure to use a condom always in every sexual encounter during a specified recall period as reported in the included studies [3,15]. The second outcome aimed to identify the associated factors of inconsistent condom use. We sought to analyze and summarize the available evidence on these associated factors to provide a comprehensive understanding of their influence on ICU.

### Eligibility criteria

To declare the inclusion and exclusion criteria in this systematic review and meta-analysis, the researchers followed a mixed framework of CoCoPop (condition: inconsistent condom use, context: African countries, population: female sex workers), and PEO (participants: female sex workers, exposure: risk factors, outcome: inconsistent condom use) [16,17].

### Inclusion criteria

The review included observational studies (cross-sectional, case-control, and cohort) conducted in African countries, both community-based and institutional-based studies that had reported the prevalence and/or associated factors with ICU.

### Exclusion criteria

Review, qualitative studies, articles with no relevant information, letters, expert opinions, different outcome measurement, absence of full text, and FSWs who identified as trans sex workers were excluded.

### Search strategy and information source

Electronic databases, including PubMed, Cochrane Library, Epistemonikos, Hinari, Science Direct, and the manual search using open Google, Google Scholar, and other African university repositories, were systematically searched. Furthermore, a search was conducted through the reference list of all identified articles to uncover additional relevant studies. The core search terms and phrases we used were prevalence, “inconsistent condom use”, “female sex workers”, “associated factors”, and “African countries”. The search was carried out from March 15–20, 2024. Boolean operators “OR” and “AND” were used to combine MeSH terms and keywords. A detailed list of the search terms used across different databases can be found in the supplementary table (**S1 Table**)

### Study selection

**Fig 1:**
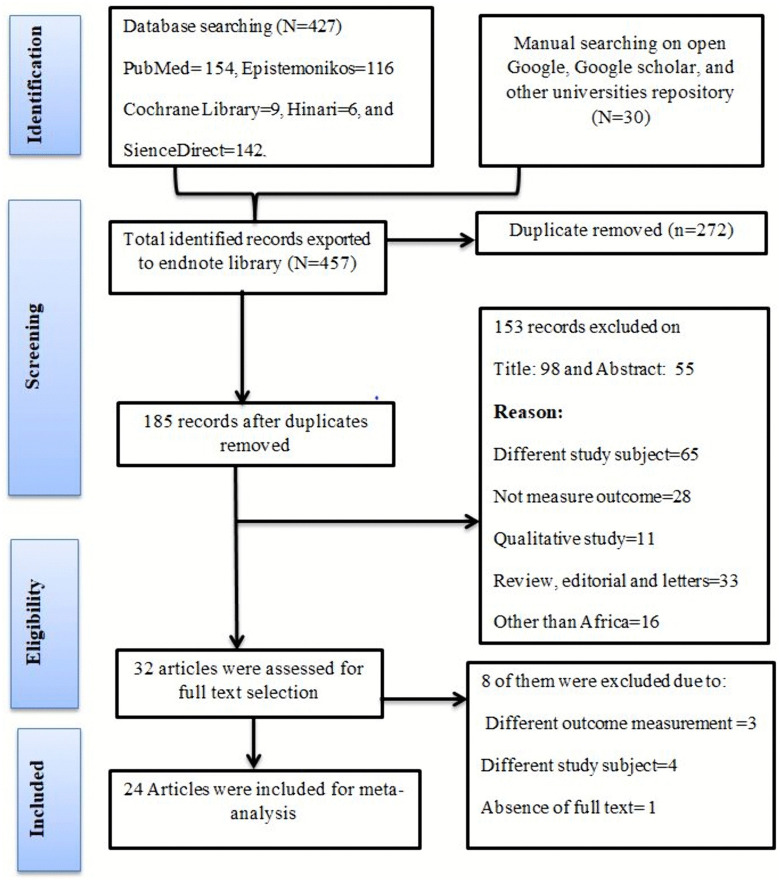
The PRISMA flow chart of selection process on inconsistent condom use among FSWs in African countries, 2025.

All 457 retrieved studies were exported to reference manager software, EndNote version 9, to remove duplicate studies, during which 272 articles were removed due to duplication. Three independent reviewers (MB, HG, and WM) screened the titles, abstracts, and full texts. The disagreements between reviewers were resolved based on established article selection criteria. Ninety-eight (98) and fifty-five (55) studies were removed through title and abstract selection, respectively. Thirty-two (32) articles were considered for full-text assessment of the eligibility, and eight of them were excluded due to the outcome variable not being measured, articles not containing relevant information, and studies being qualitative. Finally, twenty-four (24) studies were included in the pooled estimate of prevalence and/or associated factors of inconsistent condom utilization (**Fig 1**).

### Data extraction

A systematic Microsoft Excel spreadsheet data extraction form was used by two independent reviewers (MBS and WMT) to extract the data. The phase was repeated when differences in the extracted data between the reviewers were noted. If differences amongst the data extractors persisted, the third reviewer (HG) was consulted. The following information was extracted: study design, data collection method, partner type, recall period (time window of outcome measurement), study participants, sample size, prevalence of inconsistent condom utilization, country, publication year, year of study, study setting (population-based versus institutional-based), author name, and adjusted odds ratio (AOR) with a 95% confidence interval (CI) (**S3 File**).

### Risk of bias assessment

Two separate authors (MBS and WMT) assessed the risk of bias using the Joanna Briggs Institute (JBI) quality appraisal tool [18]. A third reviewer’s intervention helped to settle the dispute (HG). The following items were used to assess risk of bias among included studies: appropriate inclusion criteria; identification of confounders; strategies to handle confounders; description of study subjects and settings; set clear objective and standard criteria used; reliable and valid measurement of exposure; appropriate outcome measurement; and appropriate statistical analysis. Low-risk studies (when they scored above 50% on the quality assessment indicators) were considered for review and meta-analysis. We had 24 cross-sectional studies, and all got a score of above 50% on the quality scale, which is low risk and can be included in the study (**S2 Table**).

### Data synthesis and analysis

This systematic review and meta-analysis involved the extraction of proportion of inconsistent condom use, AOR for significant variables, and all other descriptive data, which was entered into a Microsoft Excel spreadsheet, then subsequently imported into STATA software version 17 for analysis. The data synthesis was done through a descriptive summary of the included studies via text, tables, and Figures. If the studies were appropriate for quantitative synthesis, a meta-analysis was conducted. The estimates from the studies (proportions and adjusted odds ratios) were pooled using a meta-analysis regression model to obtain an overall summary estimate. A random effect model with DerSimonian–Laird [19] estimation technique was selected for analysis because of heterogeneity that was identified in fixed effect models. To determine the presence of statistical heterogeneity, the inverse variance (I²) and Cochrane Q statistical test was used. The level of heterogeneity among the studies was quantified, and substantial heterogeneity was assumed due to I² value was greater than 75% [20]. Subgroup analysis was done by country, partner type, study year, and time window of outcome measurement. A leave one out sensitivity analysis was used to assess the influence of individual studies on the overall prevalence estimate of the meta-analysis. Publication bias was checked by the funnel plot and, more objectively, through Egger’s regression test [21]. The overall effect (pooled estimates of inconsistent condom use and factors) was reported with its proportion and adjusted odds ratio with a 95% CI.

### Ethics approval and consent to participants

Not applicable because no primary data were collected.

## Result

### Characteristics of the included studies

A total of 457 articles were retrieved from different databases: PubMed = 154, Epistemonikos = 116, Cochrane Library = 9, Hinari = 6, Science Direct = 142 and from grey literature = 30. After selection process, 24 studies were considered suitable for inclusion in this meta-analysis (**Fig 1**). All the included studies were conducted with a cross-sectional study design [3,12, 22–43] with a sample size ranging from 140 [27] to 6085 [3]. The study period (years of data collection) were conducted from 1990 to 2021 whereas, the publication years were from 1993 [37] to 2023 [41]. The reviewed studies examined inconsistent condom use among FSW with various types of sexual partners. Correspondingly, ten studies examined ICU among all type clients [22,25,26,28,31,32,38,39,41,42], six among casual clients [3,24,33,34,37,40], five among regular non-paying partners [12,23,29,30,35], and three among regular paying clients [27,36,43]. All studies were checked for risk of bias and had low risk. A total of 23,496 FSWs with a median age of 27.3 years were involved in this study. Accordingly, the magnitude of ICU was determined by pooling findings of [3,12, 22–43]. All finding is highly strengthened by (Table 1).

**Table 1:** Characteristics of studies included in the systematic review and meta-analysis of inconsistent condom use among female sex workers in Africa: 2025.

Author, Year	Study country	Study setting	Partner type	Study design	Sample Size	P (%)	Quality status
Abelson et al, 2019	Cameroon	Community based	All type	Cross sectional	2165	23.5	Low Risk
Assemahegn et al, 2015	Ethiopia	Community based	Regular nonpaying	Cross sectional	141	76.3	Low Risk
Bukenya et al, 2013	Uganda	Institution based	Casual Client	Cross sectional	905	40	Low Risk
Chabata et al, 2017	Zimbabwe	Institution based	All type	Cross sectional	1842	42	Low Risk
Decker et al, 2016	Cameroon	Community based	All type	Cross sectional	1817	59.2	Low Risk
Duff et al, 2018	Uganda	Community based	Regular nonpaying	Cross sectional	400	84.5	Low Risk
Gallo et al, 2011	Kenya	Community based	Regular paying	Cross sectional	140	64	Low Risk
Grosso et al, 2015	Gambia	Institution based	All type	Cross sectional	248	67.3	Low Risk
Josephine et al, 2013	Guinea	Community based	Regular nonpaying	Cross sectional	223	80.4	Low Risk
Kassie et al, 2008	Ethiopia	Community based	Regular nonpaying	Cross sectional	1398	70	Low Risk
Kayembe et al, 2008	Congo	Community based	All type	Cross sectional	2638	60	Low Risk
Ken Limwame et al, 2016	Malawi	Community based	All type	Cross sectional	198	54.1	Low Risk
Logie et al, 2020	Tanzania	Community based	Casual Client	Cross sectional	298	30.2	Low Risk
Minwyelet et al, 2020	Ethiopia	Community based	Casual Client	Cross sectional	307	43.4	Low Risk
Mooney et al, 2013	Ethiopia	Community based	Regular nonpaying	Cross sectional	350	38	Low Risk
Nabayinda et al, 2022	Uganda	Institution based	Regular paying	Cross sectional	426	71	Low Risk
Pickering et al, 1993	Gambia	Community based	Casual Client	Cross sectional	181	16	Low Risk
Rameto et al, 2023	Ethiopia	Community based	Casual Client	Cross sectional	6085	17.1	Low Risk
Tamene et al, 2015	Ethiopia	Community based	All type	Cross sectional	488	52.3	Low Risk
Twahirwa Rwema et al, 2019	Senegal	Community based	All type	Cross sectional	758	23.2	Low Risk
Wirtz et al, 2015	Togo &B F	Community based	Casual Client	Cross sectional	1380	23.9	Low Risk
Wondmagegn et al, 2022	Ethiopia	Community based	All type	Cross sectional	194	32.5	Low Risk
Workie et al, 2019	Ethiopia	Community based	All type	Cross sectional	156	10.3	Low Risk
Yang et al, 2020	Senegal	Community based	Regular paying	Cross sectional	758	43.4	Low Risk

P = proportion of inconsistent condom use among each included study

### Prevalence of inconsistent condom use

**Fig 2:**
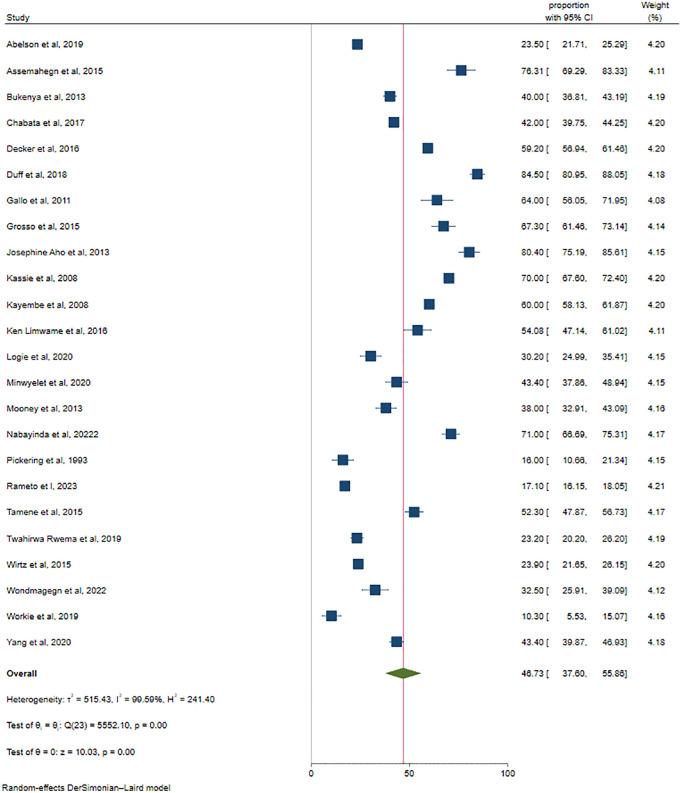
Forest plot of inconsistent condom use among female sex workers in Africa, 2025.

Computing a meta-analysis for overall inconsistent condom use with a fixed effects model resulted in a high degree of heterogeneity (Q-test = 241.40, I^2^ = 99.99%). Hence, a random-effects model with the DerSimonian-Laird method was preferred to pool the effect size for overall ICU. Accordingly, our meta-analysis resulted in the overall pooled level of ICU to be 46.73 (95% CI: 37.6, 55.86) (**Fig 2**). Given the extremely high heterogeneity among the included studies (I² ^=^ 99.59%), the pooled prevalence should be interpreted as a broad summary estimate reflecting the overall trend across studies rather than a precise prevalence applicable to every specific setting.

### Publication bias

**Fig 3:**
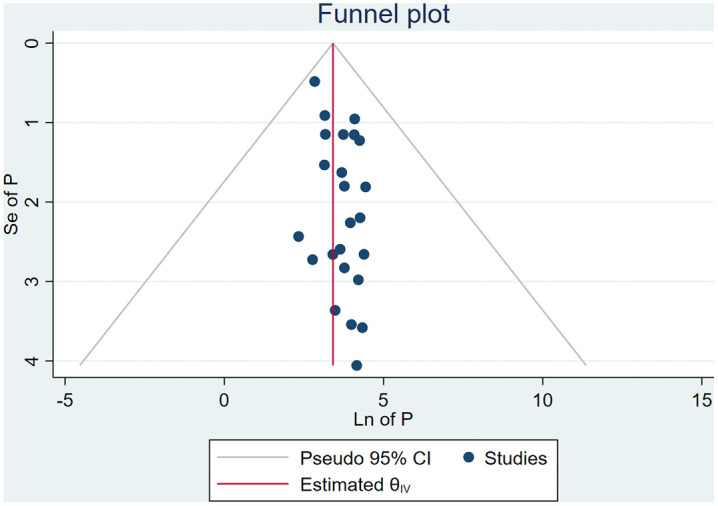
Funnel plot for inconsistent condom use among female sex workers in Africa, 2025.

The p-value for Egger’s regression test was 0.30, which indicated the absence of publication bias, and this finding is highly strengthened by the symmetrical funnel plot (**Fig 3**).

### Subgroup analysis

**Fig 4:**
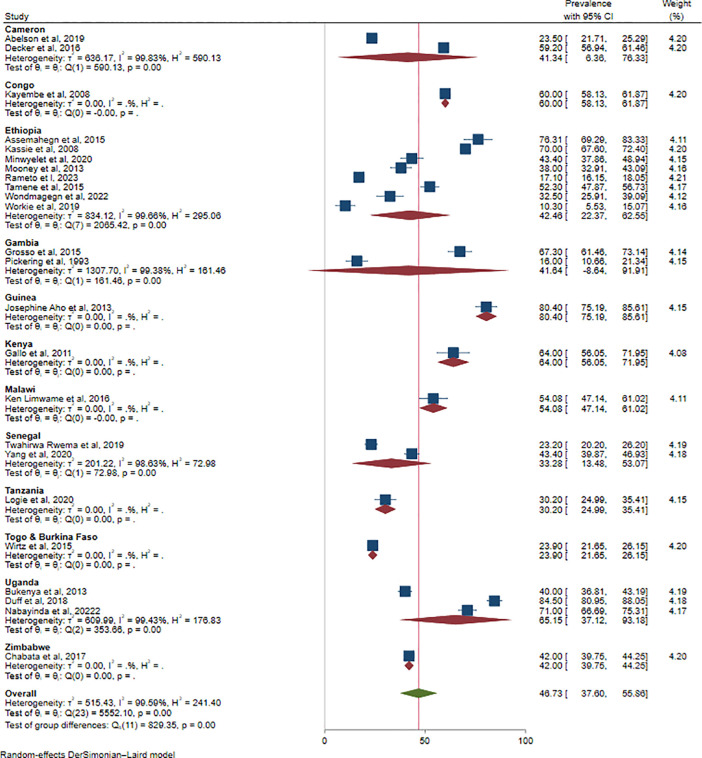
Subgroup analysis by country for inconsistent condom use among female sex workers in Africa, 2025.

As a high degree of heterogeneity presented, we performed a subgroup analysis for the pooled estimate of ICU based on country and partner type of female sex workers. Findings of subgroup analysis by country type revealed that inconsistent condom use was found to be high (80.4%) in Guinea and low (23.9%) in Togo & Burkina Faso (**Fig 4**).

**Fig 5:**
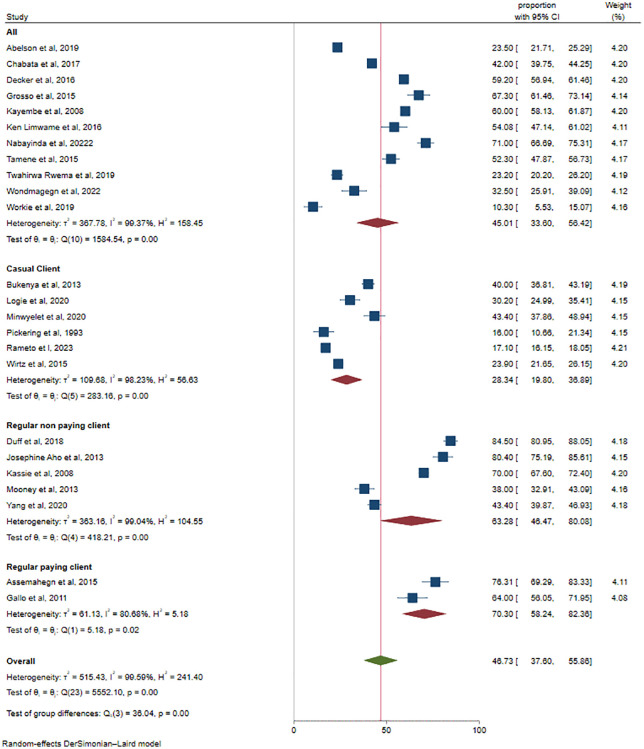
Subgroup analysis by partner type for inconsistent condom use among female sex workers in Africa, 2025.

On the other hand, the subgroup analysis was also conducted for inconsistent condom use by partner type. We found that the proportions were 45.01%, 28.34%, 63.28%, and 70.30% in all types, casual clients, regular nonpaying clients, and regular paying clients respectively (**Fig 5).**

**Fig 6:**
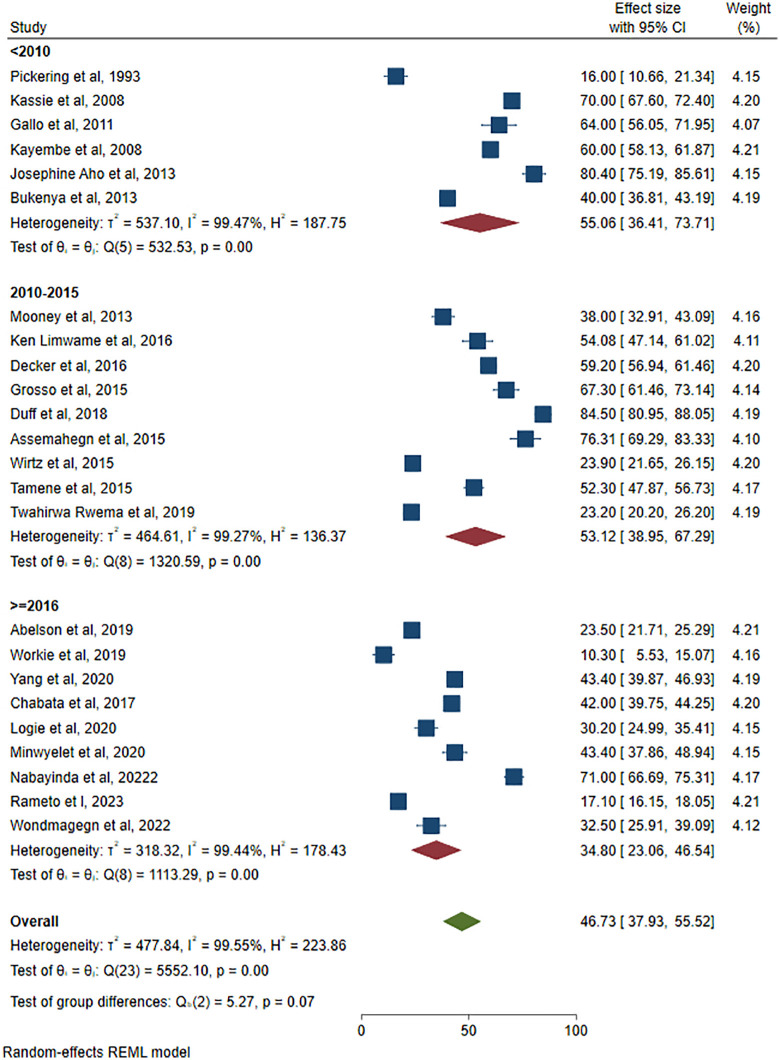
Subgroup analysis based on study years of inconsistent condom use among female sex workers in Africa, 2025.

We have also done the subgroup analysis by considering years of studies after categorizing the study years as <2010, between 2010 & 2015, and after 2016. This result showed as the pooled estimate of ICU for studies were 55.06% ((95% CI: 36.41, 73.71), I^2^ = 99.47, P = 0.00), 53.12% ((95%CI: 38.95, 67.29), I^2^ = 99.27, P = 0.00), and 34.80% ((95% CI: 23.06, 46.54), I^2^ = 99.44, P = 0.00) respectively (**Fig 6**). Despite the oldest study conducted at 1990 had the lowest prevalence due to temporal factors of the outcome variable measured (the inconsistent condom use were measured after a 14 month follow-up) the overall findings show the inconsistent condom use was decreased through time. It confirms presence of perception change of condom use among FSWs residing in Africa.

**Fig 7:**
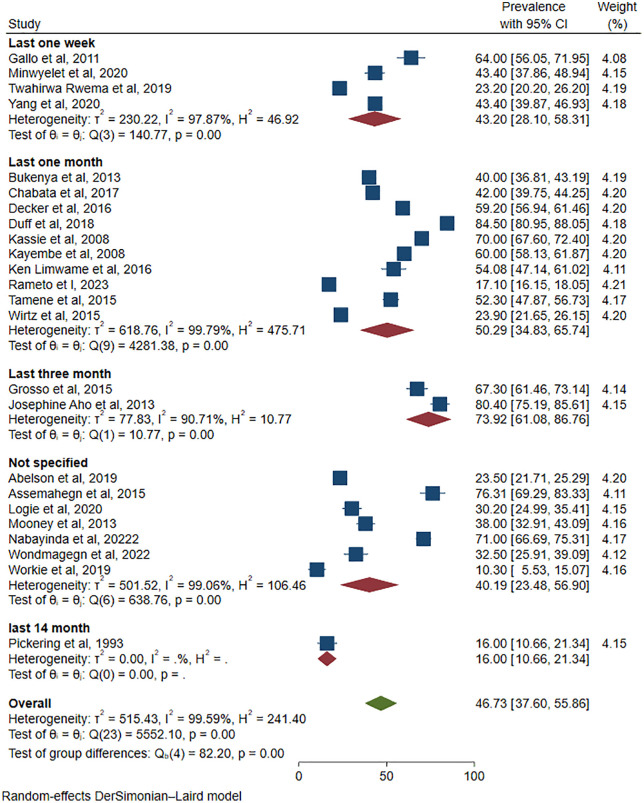
Subgroup analysis based on time window of inconsistent condom use measurement among female sex workers in Africa, 2025.

Moreover, the subgroup analysis was done based on time window of outcome measurement (Last one week, Last one month, Last three month, Last 14 month, and Not specified). Overall, 17 of the 24 studies reported a clear timeframe. There was substantial heterogeneity within each subgroup; however, the test for subgroup differences was statistically significant (Q_b_ = 82.20, p < 0.001) (**Fig 7**), indicating that the time window of measurement significantly influenced the reported prevalence of inconsistent condom use among FSWs.

### Sensitivity analysis

**Fig 8:**
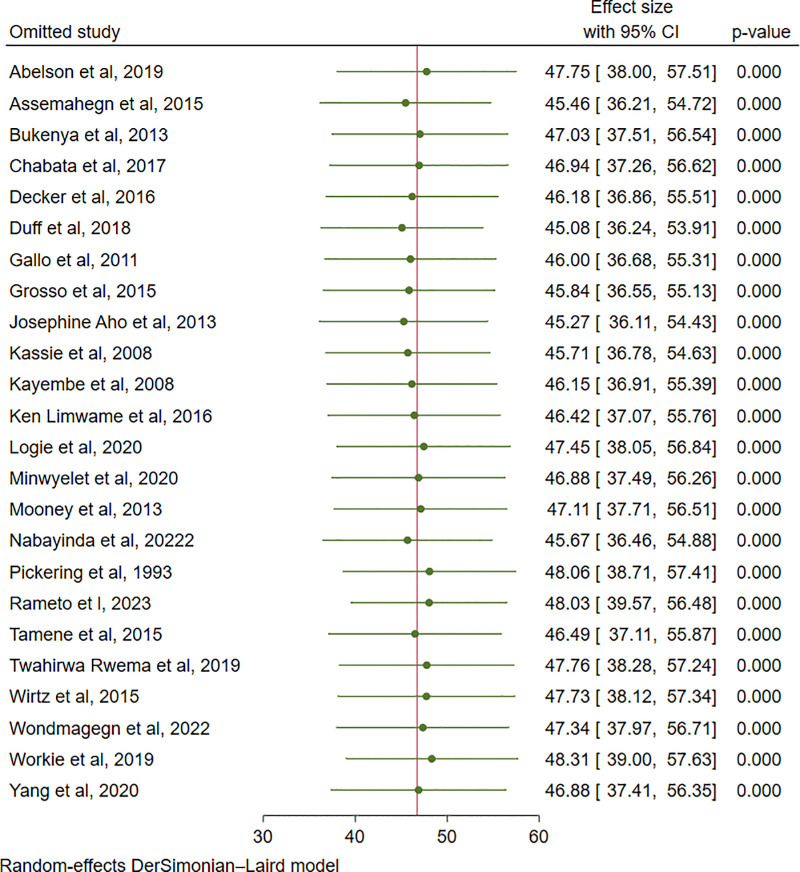
Leave-one-out sensitivity analysis for ICU among FSW in Africa, 2025.

Leave-one-out sensitivity analysis was employed to investigate the influence of each study on the overall estimate. As a result, the pooled estimates of ICU among female sex workers in Africa were steady and reliable when analyzed by omitting one study at a time (**Fig 8**).

### Meta-regression

Substantial heterogeneity was observed between the studies. To explore the sources of this heterogeneity, we conducted a meta-regression analysis, incorporating variables such as Country, partner type, sample size, study year, and recall period. However, meta-regression analysis did not reveal a significant impact on the observed variation of inconsistent condom use (**Table 2**).

**Table 2:** Shows meta-regression for inconsistent condom use among FSWs residing Africa: 2025.

Source of heterogeneity	Coefficient	Standard error	Z	P > |Z|	95% Confidence Interval
Country	−0.43	0.76	0.57	0.570	−1.06, 1.93
Partner type	4.22	4.69	0.90	0.368	−4.97, 13.42
Sample size	−4.91	9.37	−0.52	0.600	−23.28, 13.45
Study year	−1.58	1.05	−1.51	0.130	−3.63, 0.47
Recall period	−3.32	3.77	−0.88	0.378	−10.72, 4.06
Constant	63.76	28.04	2.27	0.023	8.81, 118.72

### Factors associated with inconsistent condom use

Data about nineteen different variables (alcohol use, no education, age > 20 years, > 1 year in sex work, married marital status, unfavorable knowledge on HIV, depression, drug use, violence,

perceived high HIV risk, > 9 clients number, condom availability, ≥ 2 nonpaying sexual partners, < 14 years at first sex, positive HIV status, favorable knowledge towards condom use, difficult condom negotiation, police harassment, and new clients) were extracted using an Excel sheet and analyzed separately. As a result, the pooled effect size of seven variables (condom availability, depression, no education, two or more nonpaying clients, police harassment, > 9 client’s number, and violence) showed a significant association with inconsistent condom use (**S1 File**). Accordingly, five studies were used to assess the association between condom availability and inconsistent condom use [3,28,31,39,43]. Condom availability decreased inconsistent condom use by 32% (AOR = 0.68; 95% CI: 0.50, 0.92). Three studies were included to determine the effect of depression on inconsistent condom use [3,22,25]. Female sex workers who had depression were 1.51 times more likely to use condoms inconsistently as compared with those who did not have depression (AOR = 1.51; 95% CI: 1.00, 2.30). The pooled effect of eight studies [3,23,24,32,34,35,38,43] showed that female sex workers who had no education were 1.87 times more likely to have inconsistent condom use as compared to those with secondary and above educational levels (AOR = 1.87; 95% CI: 1.19, 2.93). The combined effect of two studies [3,27] indicated that participants who had two or more regular partners, increasing the inconsistent condom use by 2.9 times compared to their counterparts (AOR = 2.90; 95% CI: 1.51, 5.54). Findings from two studies [12,33] indicated female sex workers who had a history of police harassment increased the likelihood of inconsistent condom use by a factor of 2.28 (AOR = 2.28; 95% CI: 1.03, 5.05). Similarly, the pooled effect of eight studies [3,12,22,25,26,35,38,39] showed that experiencing any form of violence increased the likelihood of inconsistent condom use by 1.74 (AOR = 1.74; 95% CI: 1.33, 2.27) times compared to those who had not experienced violence. Additionally, a synthesis of four studies [24,31,32,38] indicated that female sex workers with more than nine current clients had a 54% (AOR = 0.46; 95% CI: 0.29, 0.74) lower likelihood of ICU; all these findings were summarized below (**Table 3**).

**Table 3:** Summary estimate of AOR for factors associated with inconsistent condom use among female sex workers in Africa: 2025.

Factors	Number of studies	Effect size	95% CI	Heterogeneity (I^2^, p-value)
Condom availability	5	0.68	(0.50, 0.92)	0.00%, 0.01
Depression	3	1.51	(1.00, 2.30)	0.00%, 0.05
No education	8	1.87	(1.19, 2.93)	60.45%, 0.01
≥2 nonpaying regular client clients	2	2.90	(1.51, 5.54)	0.00%, 0.00
Police harassment	2	2.28	(1.03, 5.05)	0.00%, 0.04
Violence	8	1.74	(1.33, 2.27)	0.00%, 0.00
>9 current sexual clients	4	0.46	(0.29, 0.74)	24.62%, 0.00

## Discussion

Inconsistent condom use among female sex workers in Africa remains a critical public health concern. The overall pooled estimate of ICU among FSW in Africa was 46.73% (95% CI: 37.6, 55.86). This indicates that nearly half of FSWs in Africa had inconsistent condom use. This is consistent with a systematic review and meta-analysis of findings from low-income countries with a prevalence of 52.8% [44]. Additionally, the finding is also in line with the meta-analysis study in South America 45.9% [45].

This consistency might be due to the fact that both the low-income countries and the South American continent share similar limited access to sexual health services, and also both regions have high poverty rates, which force FSWs to choose financial survival over condom negotiation. However, the finding was higher than a study conducted in Brazil [5], and a meta-analysis study from China estimated it to be 35.2% [46]. This difference might be due to the considerable cross-country disparity, which can be contributed by socio-demographic and economic differences, including educational attainment and other health-related factors [47]. In our study, condom availability, depression, no education, having ≥2 nonpaying clients, police harassment, violence, and having ≥ 9 sexual clients had statistically significant associations with inconsistent condom use. The study indicated that condom availability decreased the odds of inconsistent condom use. This is supported by findings from Southeast Asia, which reported that access to condoms decreases the likelihood of ICU by 35% [48]. Improving the supply chains for free and easily accessible condoms in workplaces of FSWs, like hotels, and bars are critical locations. However, a significant gap in the availability of condoms for people in Africa, especially when people meet new sexual partners, has been reported [49]. Female sex workers who experience any form of violence were 1.74 times more likely to use condoms inconsistently. This finding was supported by a study from a sub-Saharan systematic and a United States scoping review [50,51]. This consistency is due to the fact that female sex workers sometimes refuse to comply with clients’ sexual demands, which can lead to violence. Additionally, many engage in sex work around bars, an environment that exposes them to multiple risks, including violence from bar matrons and forced alcohol consumption, which in turn contributes to inconsistent condom use [52]. The concerned bodies initiate strategies to address violence through policy reforms, community awareness, and legal support services. Additionally, empowering FSWs and creating a safe working environment through self-defense training could also mitigate risks. Depression increases the odds of ICU compared to their counterparts. This is congruent with studies conducted in Mexico [53]. It is due to a complex interaction of social, and psychological thoughts about dying and hopelessness about the future in FSWs who developed depression [54]. Therefore, FSWs with depression may be unable to focus on condom use and other negative thoughts and behaviors due to tiredness and poor concentration imposed by depressive symptoms [54,55]. Promoting mental health support service to FSWs is necessary. Providing counseling and stress management programs can help FSWs cope with depression and improve condom negotiation skills. Participants who had no education increased the likelihood of ICU by 1.87 times compared to FSWs who had secondary and above education. This finding was supported by evidence from a global review [45]. Expanding peer education programs for FSWs who have no education can improve them with condom use negotiation skills and increase knowledge about sexual health. FSWs that had a history of police harassment were more likely to use condoms inconsistently in this study. Our finding was in line with a scoping review conducted in sub-Saharan countries [56]. Decriminalization of sex work and reforming police practices are important. Programs that engage law enforcement in harm reduction strategies can help reduce harassment and improve public health outcomes. Female sex workers’ having nonpaying regular clients increases the odds of inconsistent condom use. This evidence is supported by different review studies [57]. Strategies focus on creating awareness about the risks of ICU with all partners, including the intimate ones. The interpretation of ICU should be considered cautiously, since the definition varies across studies depending on the time window of outcome measurement applied. The subgroup analysis confirmed that these temporal variations affected the pooled estimates of ICU. Such variability likely contributed to the substantial heterogeneity across studies.

### Strength and limitation of the study

This systematic and meta-analysis study precisely adhered to the PRISMA criteria, employed extensive search strategies, assessed the quality of the included articles, and performed a meta-analysis (subgroup analysis, leave-one-out sensitivity, meta-regression, and publication bias). However, in this review, the included studies were cross-sectional, which limited our ability to assess the cause-and-effect relationship. Despite the fact that there are many factors associated with ICU, we conducted meta-analyses only for some variables because at least two studies reporting those variables were statistically significant which required to perform the meta-analysis. Additionally, we found that heterogeneity is highly concerning, specifically on the pooled estimate of ICU, and the interpretation could be cautious. Moreover, due to the absence standardized international definition or measurement timeframe for inconsistent condoms use across studies the pooled prevalence of should be interpreted as a broad behavioral estimate rather than a precise measurement of uniform construct. Some of the included studies repot different time windows of outcome measurement and some did not report the time frame of condom use; this may challenge consistent interpretation of the inconsistent condom use in the study.

## Conclusion and recommendation

Inconsistent condom use among female sex workers in Africa remains high and a critical public health concern. This systematic review and meta-analysis indicated that violence, depression, ≥ 2 nonpaying partners, police harassment, and no education had a positive association with inconsistent condom use, whereas condom availability and have >9 current sexual client variables were negatively associated with ICU among FSWs. Long-term, routine, and population-specific condom promotion strategies should be in place to ensure condom access at all times they need. Providing mental health services like counseling and depression management is necessary. Additional strategies like women empowerment, peer education programs, and interventions on raising awareness about ICU with all partners should be implemented.

## Supporting information

S1 Table Searching strategy across all databases on inconsistent condom use among FSWs in Africa. (DOCX)

S2 Table Quality appraisal of included study for inconsistent condom use among female sex workers in Africa. (DOCX)

S1 File Pooled estimate of associated factors for inconsistent condom use among female sex workers in Africa countries. (DOCX)

S2 File PRISMA 2020 checklist for inconsistent condom use among female sex workers in Africa. (DOCX)

S3 File List of all articles, including extracted data from included studies and all excluded with their reason for exclusion on inconsistent condom use among FSWs in Africa after 272 duplication articles were removed. (XLSX)

## Abbreviations

AOR: Adjusted odds ratio
CI: Confidence interval
CoCoPop: condition, context, and population
FSWs: Female sex workers
ICU: Inconsistent condom use
HIV: Human immunodeficiency virus
PEO: participant, exposure, and outcome
PRISMA: Preferred Reporting Items for Systematic Reviews and Meta-Analyses
STI: Sexually transmitted infections
SRMA: Systematic review and meta-analysis
