# Supplementary material for: Inconsistent condom use and its associated factors among female sex workers in African countries: Systematic review and meta-analysis
Source: PLoS One. 2026 Apr 10;21(4):e0346903. doi: 10.1371/journal.pone.0346903 (PMC13068245; doi:10.1371/journal.pone.0346903)
Supplement: S1 Table — (DOCX) [file pone.0346903.s001.docx]

S1 Table: Search result for inconsistent condom use among female sex workers in Africa, 2024

| ***Mesh heading*** | ***Entry terms*** | | ***Combination*** | ***Number of article*** | ***Last Searching***  ***date*** |
| --- | --- | --- | --- | --- | --- |
|  |  |  | ***PubMed*** | | |
| **Prevalence** | proportion, magnitude | | ***((((Prevalence[Title/Abstract] OR Proportion[Title/Abstract] OR Magnitude[Title/Abstract]) AND ("Inconsistent condom use"[Title/Abstract] OR "Unprotected sex"[Title/Abstract] OR "Condom less sex"[Title/Abstract] OR "Condom use"[Title/Abstract])) AND ("Female sex worker*"[Title/Abstract] OR FSW[Title/Abstract] OR "Sex worker*"[Title/Abstract] OR "sex worker client*"[Title/Abstract] OR prostitute*[Title/Abstract] OR "female prostitute*"[Title/Abstract] OR "sex trade"[Title/Abstract] OR "sex trade worker*"[Title/Abstract] OR "transactional sex"[Title/Abstract] OR "exchange sex"[Title/Abstract] OR "fish-for-sex"[Title/Abstract] OR "commercial sex"[Title/Abstract] OR "commercial sex worker*"[Title/Abstract])) AND (Africa[Title/Abstract] OR Ethiopia[Title/Abstract] OR "South Africa"[Title/Abstract] OR Nigeria[Title/Abstract] OR Kenya[Title/Abstract] OR Ghana[Title/Abstract] OR Congo[Title/Abstract] OR Morocco[Title/Abstract] OR Tanzania[Title/Abstract] OR Senegal[Title/Abstract] OR Uganda[Title/Abstract] OR Mali[Title/Abstract] OR Cameron[Title/Abstract] OR Rwanda[Title/Abstract] OR "South Sudan"[Title/Abstract] OR Sudan[Title/Abstract] OR Niger[Title/Abstract] OR Madagascar[Title/Abstract] OR Somalia[Title/Abstract] OR "Cote d'ivoire"[Title/Abstract] OR Algeria[Title/Abstract] OR Angola[Title/Abstract] OR Zimbabwe[Title/Abstract] OR Namibia[Title/Abstract] OR Tunisia[Title/Abstract] OR Gambia[Title/Abstract] OR "Burkina Faso"[Title/Abstract] OR Guinea[Title/Abstract] OR Mozambique[Title/Abstract] OR Zambia[Title/Abstract] OR Libya[Title/Abstract] OR Chad[Title/Abstract] OR Mauritania[Title/Abstract] OR Togo[Title/Abstract] OR Liberia[Title/Abstract] OR Botswana[Title/Abstract] OR "Sierra Leon"[Title/Abstract] OR Gabon[Title/Abstract] OR "Central African Republic"[Title/Abstract] OR Burundi[Title/Abstract] OR Malawi[Title/Abstract] OR Mauritius[Title/Abstract] OR "Equatorial Guinea"[Title/Abstract] OR Benin[Title/Abstract] OR Eritrea[Title/Abstract] OR Djibouti[Title/Abstract] OR Lesotho[Title/Abstract] OR Seychelles[Title/Abstract] OR "Western Sahara"[Title/Abstract])) AND ("Associated factors"[Title/Abstract] OR Determinants[Title/Abstract] OR factors[Title/Abstract] OR predictors[Title/Abstract])*** | ***154*** | ***20/03/2024*** |
| **Inconsistent condom use** | Unprotected sex, condom less sex, condom use | |  |  |  |
| **Female sex workers** | FSW, “Sex workers”, “sex worker clients”, prostitutes “female prostitutes”, “sex trade”, “sex trade workers”, “transactional sex”, “exchange sex”, “fish-for-sex” “commercial sex”, “commercial sex worker”, | |  |  |  |
| ***Africa*** | Ethiopia, "South Africa", Nigeria, Kenya, Ghana, Congo, Morocco, Tanzania, Senegal, Uganda, Mali, Cameron, Rwanda, "South Sudan", Sudan, Niger, Madagascar, Somalia, "Cote d'ivoire", Algeria, Angola, Zimbabwe, Namibia, Tunisia, Gambia, "Burkina Faso", Guinea, Mozambique, Zambia, Libya, Chad, Mauritania, Togo, Liberia, Botswana, "Sierra Leon", Gabon, "Central African Republic", Burundi, Malawi, Mauritius, "Equatorial Guinea", Benin, Eritrea, Djibouti, Lesotho, Seychelles, "Western Sahara” | |  |  |  |
| ***Associated factors*** | ***Determinants, factors, predictors*** | |  |  |  |
| ***Epistemonikos*** | | | | | |
| (title:((title:((title:(Prevalence OR Proportion OR Magnitude) OR abstract:(Prevalence OR Proportion OR Magnitude)) AND (title:("Inconsistent condom use" OR "Unprotected sex" OR "Condom less sex" OR "Condom use") OR abstract:("Inconsistent condom use" OR "Unprotected sex" OR "Condom less sex" OR "Condom use")) AND (title:("Female sex workers" OR FSW OR "Sex workers" OR "sex worker clients" OR prostitutes OR "female prostitutes" OR "sex trade" OR "sex trade workers" OR "transactional sex" OR "exchange sex" OR "fish-for-sex" OR "commercial sex" OR "commercial sex worker") OR abstract:("Female sex workers" OR FSW OR "Sex workers" OR "sex worker clients" OR prostitutes OR "female prostitutes" OR "sex trade" OR "sex trade workers" OR "transactional sex" OR "exchange sex" OR "fish-for-sex" OR "commercial sex" OR "commercial sex worker")) AND (title:(Africa OR Ethiopia OR "South Africa" OR Nigeria OR Kenya OR Ghana OR Congo OR Morocco OR Tanzania OR Senegal OR Uganda OR Mali OR Cameron OR Rwanda OR "South Sudan" OR Sudan OR Niger OR Madagascar OR Somalia OR "Cote d'ivoire" OR Algeria OR Angola OR Zimbabwe OR Namibia OR Tunisia OR Gambia OR "Burkina Faso" OR Guinea OR Mozambique OR Zambia OR Libya OR Chad OR Mauritania OR Togo OR Liberia OR Botswana OR "Sierra Leon" OR Gabon OR "Central African Republic" OR Burundi OR Malawi OR Mauritius OR "Equatorial Guinea" OR Benin OR Eritrea OR Djibouti OR Lesotho OR Seychelles OR "Western Sahara") OR abstract:(Africa OR Ethiopia OR "South Africa" OR Nigeria OR Kenya OR Ghana OR Congo OR Morocco OR Tanzania OR Senegal OR Uganda OR Mali OR Cameron OR Rwanda OR "South Sudan" OR Sudan OR Niger OR Madagascar OR Somalia OR "Cote d'ivoire" OR Algeria OR Angola OR Zimbabwe OR Namibia OR Tunisia OR Gambia OR "Burkina Faso" OR Guinea OR Mozambique OR Zambia OR Libya OR Chad OR Mauritania OR Togo OR Liberia OR Botswana OR "Sierra Leon" OR Gabon OR "Central African Republic" OR Burundi OR Malawi OR Mauritius OR "Equatorial Guinea" OR Benin OR Eritrea OR Djibouti OR Lesotho OR Seychelles OR "Western Sahara")) AND (title:("Associated factors" OR Determinants OR factors OR predictors) OR abstract:("Associated factors" OR Determinants OR factors OR predictors))) OR abstract:((title:(Prevalence OR Proportion OR Magnitude) OR abstract:(Prevalence OR Proportion OR Magnitude)) AND (title:("Inconsistent condom use" OR "Unprotected sex" OR "Condom less sex" OR "Condom use") OR abstract:("Inconsistent condom use" OR "Unprotected sex" OR "Condom less sex" OR "Condom use")) AND (title:("Female sex workers" OR FSW OR "Sex workers" OR "sex worker clients" OR prostitutes OR "female prostitutes" OR "sex trade" OR "sex trade workers" OR "transactional sex" OR "exchange sex" OR "fish-for-sex" OR "commercial sex" OR "commercial sex worker") OR abstract:("Female sex workers" OR FSW OR "Sex workers" OR "sex worker clients" OR prostitutes OR "female prostitutes" OR "sex trade" OR "sex trade workers" OR "transactional sex" OR "exchange sex" OR "fish-for-sex" OR "commercial sex" OR "commercial sex worker")) AND (title:(Africa OR Ethiopia OR "South Africa" OR Nigeria OR Kenya OR Ghana OR Congo OR Morocco OR Tanzania OR Senegal OR Uganda OR Mali OR Cameron OR Rwanda OR "South Sudan" OR Sudan OR Niger OR Madagascar OR Somalia OR "Cote d'ivoire" OR Algeria OR Angola OR Zimbabwe OR Namibia OR Tunisia OR Gambia OR "Burkina Faso" OR Guinea OR Mozambique OR Zambia OR Libya OR Chad OR Mauritania OR Togo OR Liberia OR Botswana OR "Sierra Leon" OR Gabon OR "Central African Republic" OR Burundi OR Malawi OR Mauritius OR "Equatorial Guinea" OR Benin OR Eritrea OR Djibouti OR Lesotho OR Seychelles OR "Western Sahara") OR abstract:(Africa OR Ethiopia OR "South Africa" OR Nigeria OR Kenya OR Ghana OR Congo OR Morocco OR Tanzania OR Senegal OR Uganda OR Mali OR Cameron OR Rwanda OR "South Sudan" OR Sudan OR Niger OR Madagascar OR Somalia OR "Cote d'ivoire" OR Algeria OR Angola OR Zimbabwe OR Namibia OR Tunisia OR Gambia OR "Burkina Faso" OR Guinea OR Mozambique OR Zambia OR Libya OR Chad OR Mauritania OR Togo OR Liberia OR Botswana OR "Sierra Leon" OR Gabon OR "Central African Republic" OR Burundi OR Malawi OR Mauritius OR "Equatorial Guinea" OR Benin OR Eritrea OR Djibouti OR Lesotho OR Seychelles OR "Western Sahara")) AND (title:("Associated factors" OR Determinants OR factors OR predictors) OR abstract:("Associated factors" OR Determinants OR factors OR predictors))))) OR abstract:((title:((title:(Prevalence OR Proportion OR Magnitude) OR abstract:(Prevalence OR Proportion OR Magnitude)) AND (title:("Inconsistent condom use" OR "Unprotected sex" OR "Condom less sex" OR "Condom use") OR abstract:("Inconsistent condom use" OR "Unprotected sex" OR "Condom less sex" OR "Condom use")) AND (title:("Female sex workers" OR FSW OR "Sex workers" OR "sex worker clients" OR prostitutes OR "female prostitutes" OR "sex trade" OR "sex trade workers" OR "transactional sex" OR "exchange sex" OR "fish-for-sex" OR "commercial sex" OR "commercial sex worker") OR abstract:("Female sex workers" OR FSW OR "Sex workers" OR "sex worker clients" OR prostitutes OR "female prostitutes" OR "sex trade" OR "sex trade workers" OR "transactional sex" OR "exchange sex" OR "fish-for-sex" OR "commercial sex" OR "commercial sex worker")) AND (title:(Africa OR Ethiopia OR "South Africa" OR Nigeria OR Kenya OR Ghana OR Congo OR Morocco OR Tanzania OR Senegal OR Uganda OR Mali OR Cameron OR Rwanda OR "South Sudan" OR Sudan OR Niger OR Madagascar OR Somalia OR "Cote d'ivoire" OR Algeria OR Angola OR Zimbabwe OR Namibia OR Tunisia OR Gambia OR "Burkina Faso" OR Guinea OR Mozambique OR Zambia OR Libya OR Chad OR Mauritania OR Togo OR Liberia OR Botswana OR "Sierra Leon" OR Gabon OR "Central African Republic" OR Burundi OR Malawi OR Mauritius OR "Equatorial Guinea" OR Benin OR Eritrea OR Djibouti OR Lesotho OR Seychelles OR "Western Sahara") OR abstract:(Africa OR Ethiopia OR "South Africa" OR Nigeria OR Kenya OR Ghana OR Congo OR Morocco OR Tanzania OR Senegal OR Uganda OR Mali OR Cameron OR Rwanda OR "South Sudan" OR Sudan OR Niger OR Madagascar OR Somalia OR "Cote d'ivoire" OR Algeria OR Angola OR Zimbabwe OR Namibia OR Tunisia OR Gambia OR "Burkina Faso" OR Guinea OR Mozambique OR Zambia OR Libya OR Chad OR Mauritania OR Togo OR Liberia OR Botswana OR "Sierra Leon" OR Gabon OR "Central African Republic" OR Burundi OR Malawi OR Mauritius OR "Equatorial Guinea" OR Benin OR Eritrea OR Djibouti OR Lesotho OR Seychelles OR "Western Sahara")) AND (title:("Associated factors" OR Determinants OR factors OR predictors) OR abstract:("Associated factors" OR Determinants OR factors OR predictors))) OR abstract:((title:(Prevalence OR Proportion OR Magnitude) OR abstract:(Prevalence OR Proportion OR Magnitude)) AND (title:("Inconsistent condom use" OR "Unprotected sex" OR "Condom less sex" OR "Condom use") OR abstract:("Inconsistent condom use" OR "Unprotected sex" OR "Condom less sex" OR "Condom use")) AND (title:("Female sex workers" OR FSW OR "Sex workers" OR "sex worker clients" OR prostitutes OR "female prostitutes" OR "sex trade" OR "sex trade workers" OR "transactional sex" OR "exchange sex" OR "fish-for-sex" OR "commercial sex" OR "commercial sex worker") OR abstract:("Female sex workers" OR FSW OR "Sex workers" OR "sex worker clients" OR prostitutes OR "female prostitutes" OR "sex trade" OR "sex trade workers" OR "transactional sex" OR "exchange sex" OR "fish-for-sex" OR "commercial sex" OR "commercial sex worker")) AND (title:(Africa OR Ethiopia OR "South Africa" OR Nigeria OR Kenya OR Ghana OR Congo OR Morocco OR Tanzania OR Senegal OR Uganda OR Mali OR Cameron OR Rwanda OR "South Sudan" OR Sudan OR Niger OR Madagascar OR Somalia OR "Cote d'ivoire" OR Algeria OR Angola OR Zimbabwe OR Namibia OR Tunisia OR Gambia OR "Burkina Faso" OR Guinea OR Mozambique OR Zambia OR Libya OR Chad OR Mauritania OR Togo OR Liberia OR Botswana OR "Sierra Leon" OR Gabon OR "Central African Republic" OR Burundi OR Malawi OR Mauritius OR "Equatorial Guinea" OR Benin OR Eritrea OR Djibouti OR Lesotho OR Seychelles OR "Western Sahara") OR abstract:(Africa OR Ethiopia OR "South Africa" OR Nigeria OR Kenya OR Ghana OR Congo OR Morocco OR Tanzania OR Senegal OR Uganda OR Mali OR Cameron OR Rwanda OR "South Sudan" OR Sudan OR Niger OR Madagascar OR Somalia OR "Cote d'ivoire" OR Algeria OR Angola OR Zimbabwe OR Namibia OR Tunisia OR Gambia OR "Burkina Faso" OR Guinea OR Mozambique OR Zambia OR Libya OR Chad OR Mauritania OR Togo OR Liberia OR Botswana OR "Sierra Leon" OR Gabon OR "Central African Republic" OR Burundi OR Malawi OR Mauritius OR "Equatorial Guinea" OR Benin OR Eritrea OR Djibouti OR Lesotho OR Seychelles OR "Western Sahara")) AND (title:("Associated factors" OR Determinants OR factors OR predictors) OR abstract:("Associated factors" OR Determinants OR factors OR predictors)))))) | | | | ***116*** | ***20/03/2024*** |
| ***Hinari*** | | | | | |
| (TitleCombined:(Prevalence OR Proportion OR Magnitude)) AND (TitleCombined:(“Inconsistent condom use” OR “Unprotected sex” OR “Condom less sex” OR “Condom use”)) AND (TitleCombined:(“Female sex workers” OR FSW OR “Sex workers” OR “sex worker clients” OR prostitutes OR “female prostitutes” OR “sex trade” OR “sex trade workers” OR “transactional sex” OR “exchange sex” OR “fish-for-sex” OR “commercial sex” OR “commercial sex workers”)) AND (TitleCombined:(“Associated factors” OR Determinants OR factors OR predictors)) | | | | ***6*** | ***20/03/2024*** |
| **Science Direct** | | | | | |
|  | | ((“Inconsistent condom use” OR “Unprotected sex”) AND (“Female sex workers” OR prostitutes OR "exchange sex" OR “ commercial sex workers” OR “transactional sex” OR “sex trade) AND (Africa)) | | ***142*** | ***20/03/2024*** |
|  | |  | |  |  |
| ***Cochrane library*** | | | | | |
|  | | Prevalence OR Proportion OR Magnitude in Title Abstract Keyword AND “Inconsistent condom use” OR “Unprotected sex” OR “Condom less sex” OR “Condom use” in Title Abstract Keyword AND “Female sex workers” OR FSW OR “Sex workers” OR “sex worker clients” OR prostitutes OR “female prostitutes” OR “sex trade” OR “sex trade workers” OR “transactional sex” OR “exchange sex” OR “fish-for-sex” OR “commercial sex” OR “commercial sex workers” in Title Abstract Keyword AND “Associated factors” OR Determinants OR factors OR predictors in Title Abstract Keyword AND Africa in All Text - (Word variations have been searched) | | ***9*** | ***20/03/2024*** |
|  | | ***From Grey literature(Google scholar, Goggle, and African university repositories*** | | ***30*** | ***20/12/2024*** |
