# Supplementary material for: Inconsistent condom use and its associated factors among female sex workers in African countries: Systematic review and meta-analysis
Source: PLoS One. 2026 Apr 10;21(4):e0346903. doi: 10.1371/journal.pone.0346903 (PMC13068245; doi:10.1371/journal.pone.0346903)
Supplement: S2 Table — (DOCX) [file pone.0346903.s002.docx]

S2 Table: Quality appraisal of included study for inconsistent condom use among female sex workers in Africa, 2024

| Included studies | Eight JBI Critical Appraisal Checklist for Cross Sectional Studies(Yes, No, Unclear)  If Yes(>=50%).....low risk(two raters).....>1=Yes 2=No 3= Unclear | | | | | | | | | | | | | | | | |
| --- | --- | --- | --- | --- | --- | --- | --- | --- | --- | --- | --- | --- | --- | --- | --- | --- | --- |
|  | Appropriate Inclusion criteria | | Description of study subject and setting | | Valid and reliable measurement of exposure | | Objective and standard criteria used | | Identification of confounder | | Strategies to handle confounder | | Appropriate Outcome measurement | | Appropriate statistical analysis | | Over all appraisal |
|  | R1 | R2 | R1 | R2 | R1 | R2 | R1 | R2 | R1 | R2 | R1 | R2 | R1 | R2 | R1 | R2 |  |
| 1.Abelson et al | 1 | 1 | 1 | 1 | 1 | 2 | 2 | 2 | 1 | 1 | 1 | 1 | 1 | 1 | 1 | 1 | 0.8125 |
| 2.Assemahegn | 2 | 2 | 1 | 1 | 1 | 1 | 1 | 1 | 3 | 3 | 1 | 1 | 1 | 1 | 1 | 1 | 0.75 |
| 3.Bukenya et al | 1 | 1 | 1 | 1 | 1 | 1 | 1 | 1 | 2 | 2 | 2 | 1 | 1 | 1 | 1 | 1 | 0.8125 |
| 4.Chabata et al | 1 | 1 | 1 | 1 | 1 | 1 | 1 | 1 | 2 | 2 | 2 | 1 | 1 | 1 | 1 | 1 | 0.8125 |
| 5.Decker et al | 1 | 2 | 1 | 1 | 1 | 1 | 1 | 1 | 1 | 1 | 1 | 1 | 1 | 1 | 1 | 1 | 0.9375 |
| 6.Duff et al | 1 | 1 | 1 | 1 | 1 | 1 | 1 | 1 | 2 | 2 | 2 | 3 | 1 | 1 | 1 | 1 | 0.8125 |
| 7.Gallo et al | 2 | 2 | 1 | 1 | 1 | 1 | 1 | 1 | 2 | 2 | 1 | 3 | 1 | 1 | 1 | 1 | 0.6875 |
| 8.Grosso et al | 1 | 1 | 1 | 1 | 1 | 1 | 1 | 1 | 3 | 3 | 3 | 3 | 1 | 1 | 1 | 1 | 0.75 |
| 9.Josephine et al | 1 | 1 | 1 | 1 | 1 | 1 | 1 | 1 | 2 | 2 | 2 | 2 | 1 | 1 | 1 | 1 | 0.75 |
| 10.Kassie et al | 1 | 1 | 1 | 1 | 3 | 1 | 3 | 1 | 1 | 1 | 1 | 1 | 1 | 1 | 1 | 1 | 0.875 |
| 11.Kayembe et al | 1 | 1 | 1 | 1 | 1 | 1 | 1 | 1 | 3 | 3 | 2 | 1 | 1 | 1 | 1 | 1 | 0.8125 |
| 12.Ken Limwam | 1 | 1 | 1 | 1 | 1 | 1 | 1 | 1 | 3 | 3 | 2 | 1 | 1 | 1 | 1 | 1 | 0.8125 |
| 13.Logie et al | 1 | 1 | 1 | 1 | 1 | 1 | 1 | 1 | 3 | 3 | 3 | 1 | 1 | 1 | 1 | 1 | 0.8125 |
| 14.Minwyelet | 2 | 2 | 1 | 1 | 1 | 1 | 1 | 1 | 2 | 1 | 2 | 1 | 1 | 1 | 1 | 1 | 0.75 |
| 15.Mooney et al | 1 | 1 | 1 | 1 | 1 | 1 | 1 | 2 | 2 | 2 | 1 | 1 | 1 | 1 | 1 | 1 | 0.8125 |
| 16.Nabayinda | 2 | 2 | 1 | 1 | 1 | 1 | 1 | 3 | 3 | 3 | 3 | 1 | 1 | 1 | 1 | 1 | 0.625 |
| 17.Pickering et al | 1 | 1 | 1 | 1 | 1 | 1 | 2 | 1 | 1 | 2 | 1 | 1 | 1 | 1 | 1 | 1 | 0.875 |
| 18.Rameto et al | 1 | 1 | 1 | 1 | 1 | 1 | 1 | 1 | 1 | 2 | 1 | 1 | 1 | 1 | 1 | 1 | 0.9375 |
| 19.Tamene et al | 1 | 2 | 1 | 1 | 1 | 1 | 1 | 2 | 1 | 1 | 2 | 1 | 1 | 1 | 1 | 1 | 0.8125 |
| 20.Twahirwa et al | 1 | 1 | 1 | 1 | 1 | 3 | 1 | 1 | 1 | 1 | 1 | 1 | 1 | 1 | 1 | 1 | 0.9375 |
| 21.Wirtz et al | 1 | 1 | 1 | 1 | 1 | 1 | 2 | 3 | 1 | 1 | 1 | 1 | 1 | 1 | 1 | 1 | 0.875 |
| 22.Wondmagegn | 2 | 1 | 1 | 1 | 1 | 1 | 1 | 2 | 1 | 1 | 1 | 3 | 1 | 1 | 1 | 1 | 0.8125 |
| 23.Workie et al | 1 | 1 | 1 | 1 | 2 | 2 | 1 | 1 | 1 | 1 | 1 | 1 | 1 | 1 | 1 | 1 | 0.875 |
| 24.Yang et al | 1 | 1 | 1 | 1 | 1 | 1 | 1 | 1 | 1 | 2 | 1 | 1 | 1 | 1 | 1 | 1 | 0.9375 |
