# Supplementary material for: Inconsistent condom use and its associated factors among female sex workers in African countries: Systematic review and meta-analysis
Source: PLoS One. 2026 Apr 10;21(4):e0346903. doi: 10.1371/journal.pone.0346903 (PMC13068245; doi:10.1371/journal.pone.0346903)

**S1 File:** Pooled estimate of associated factors for inconsistent condom use among female sex workers in Africa countries


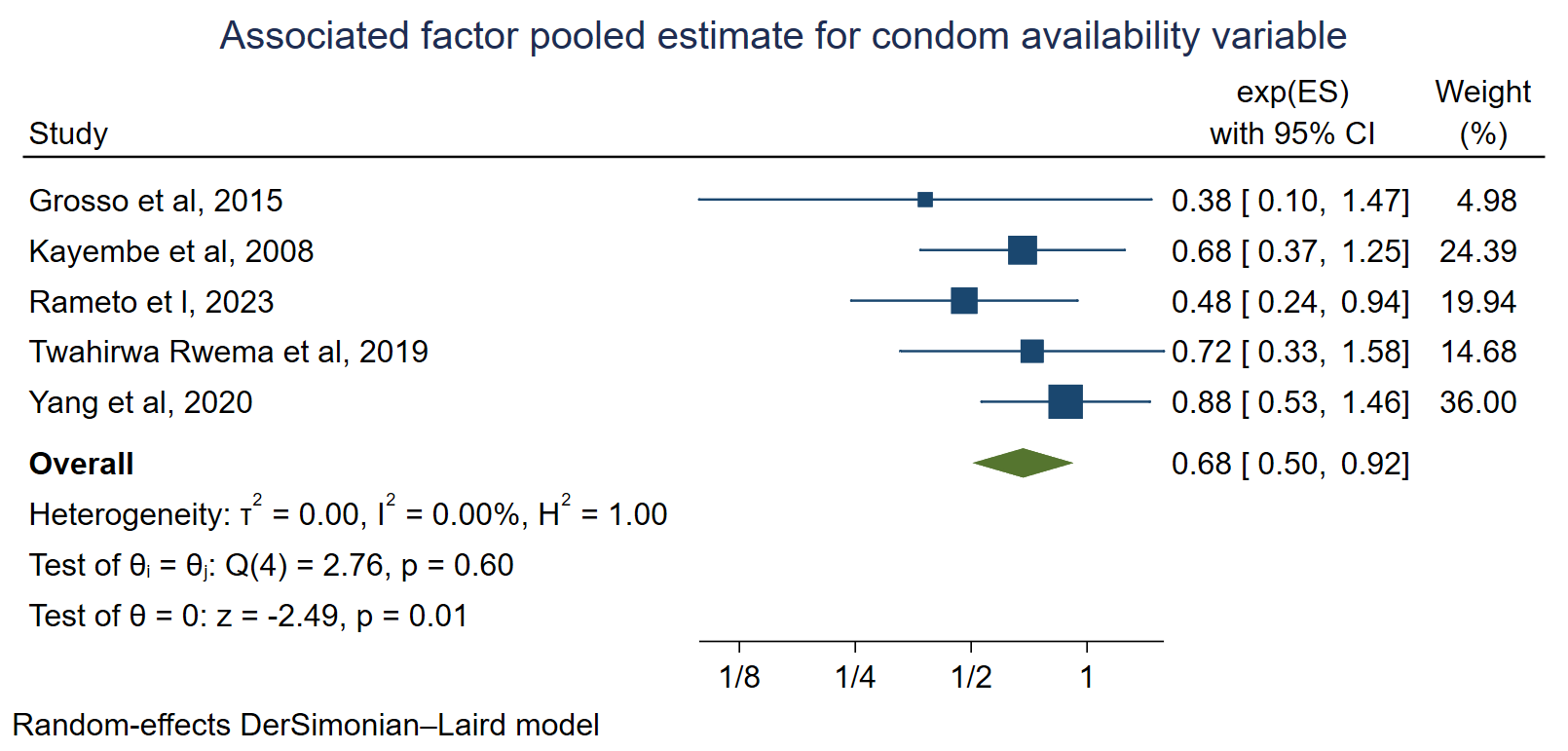


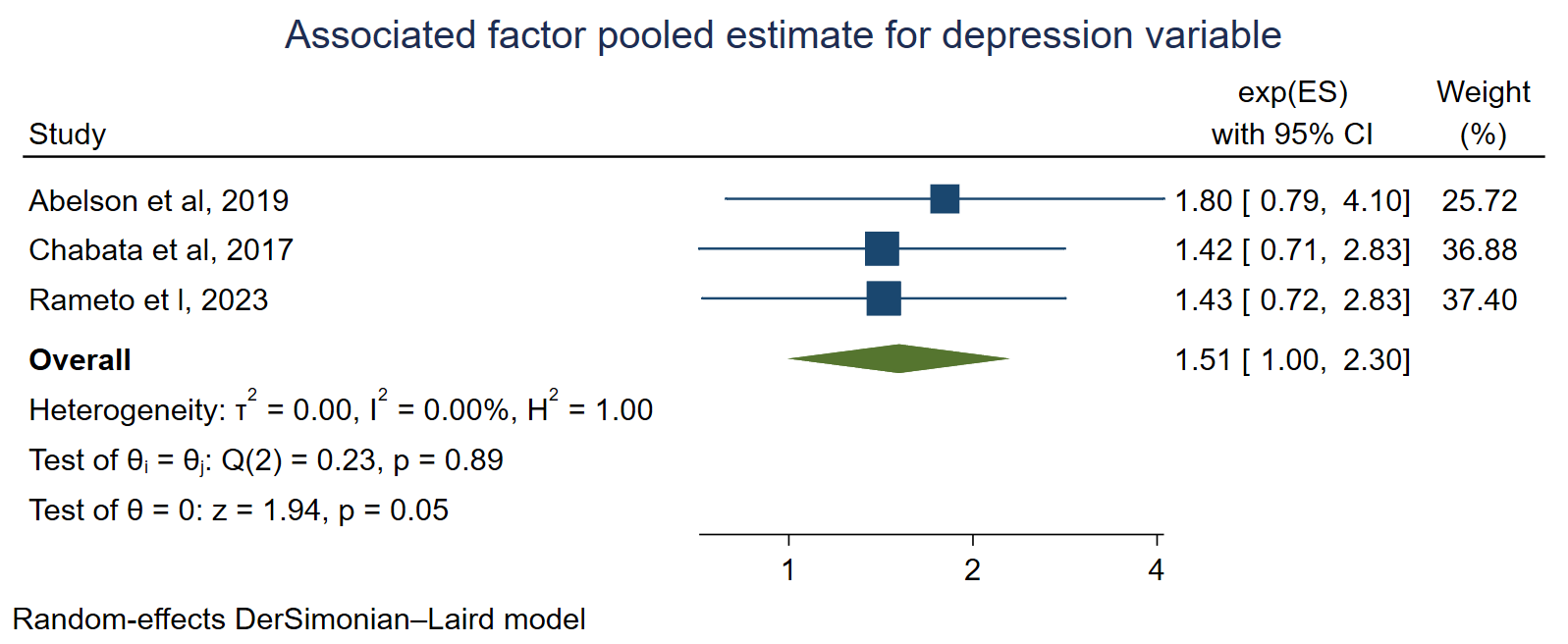


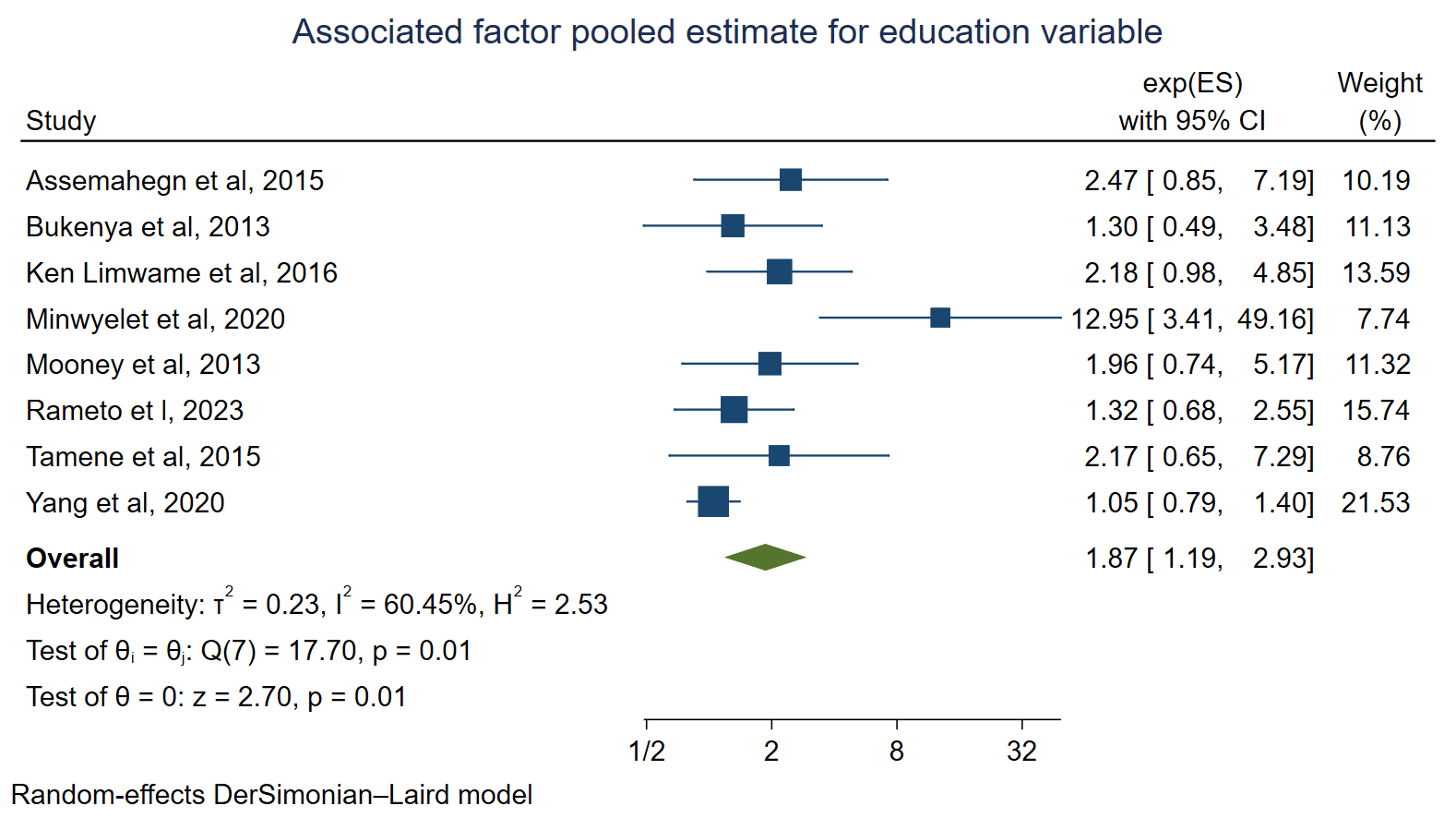


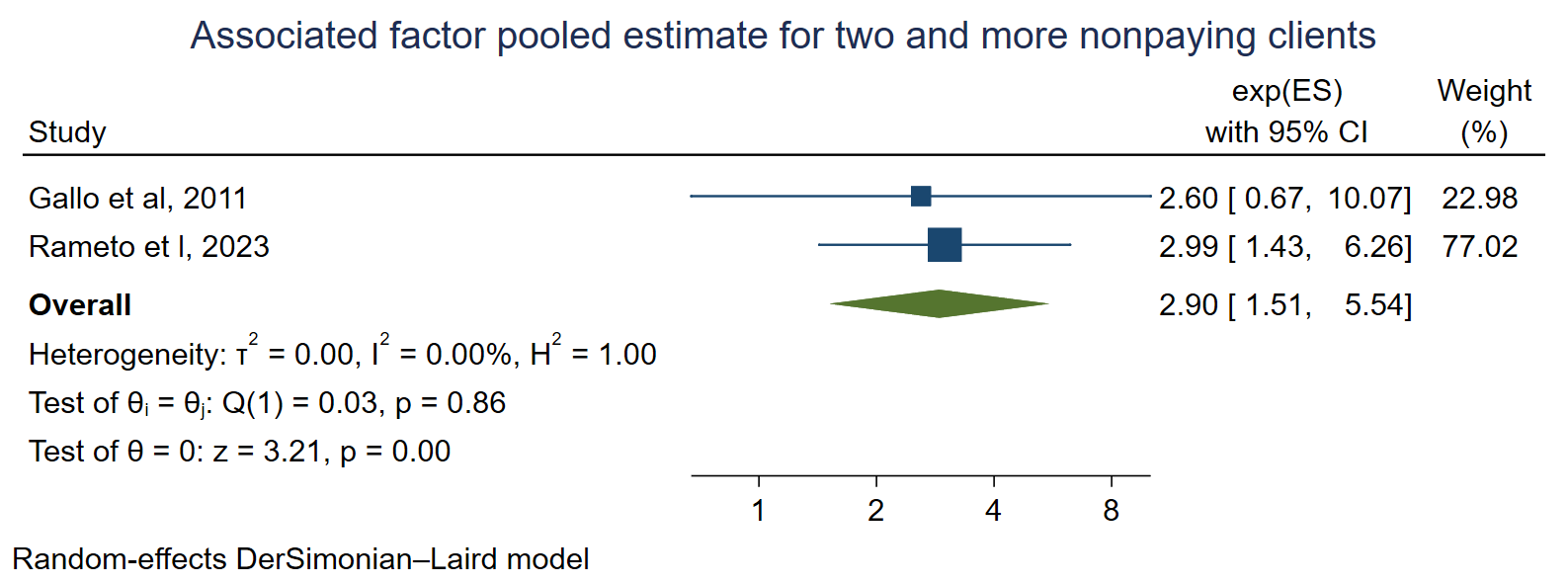


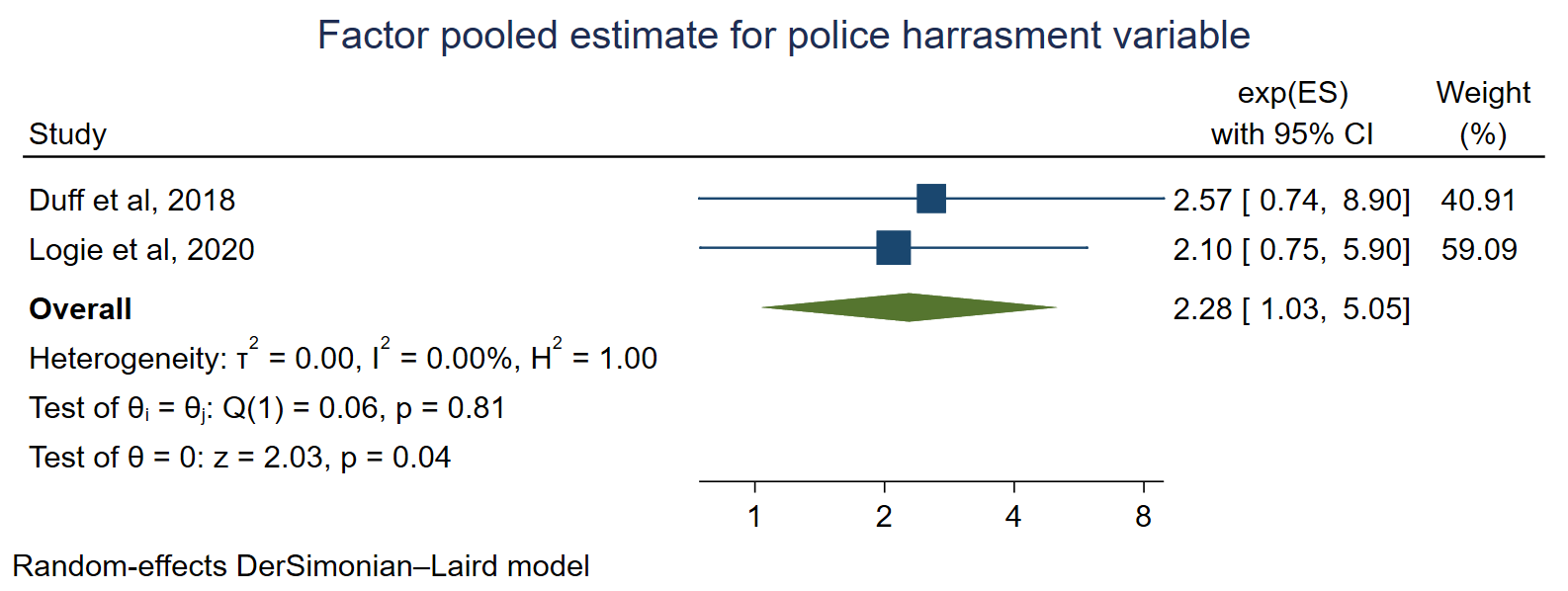


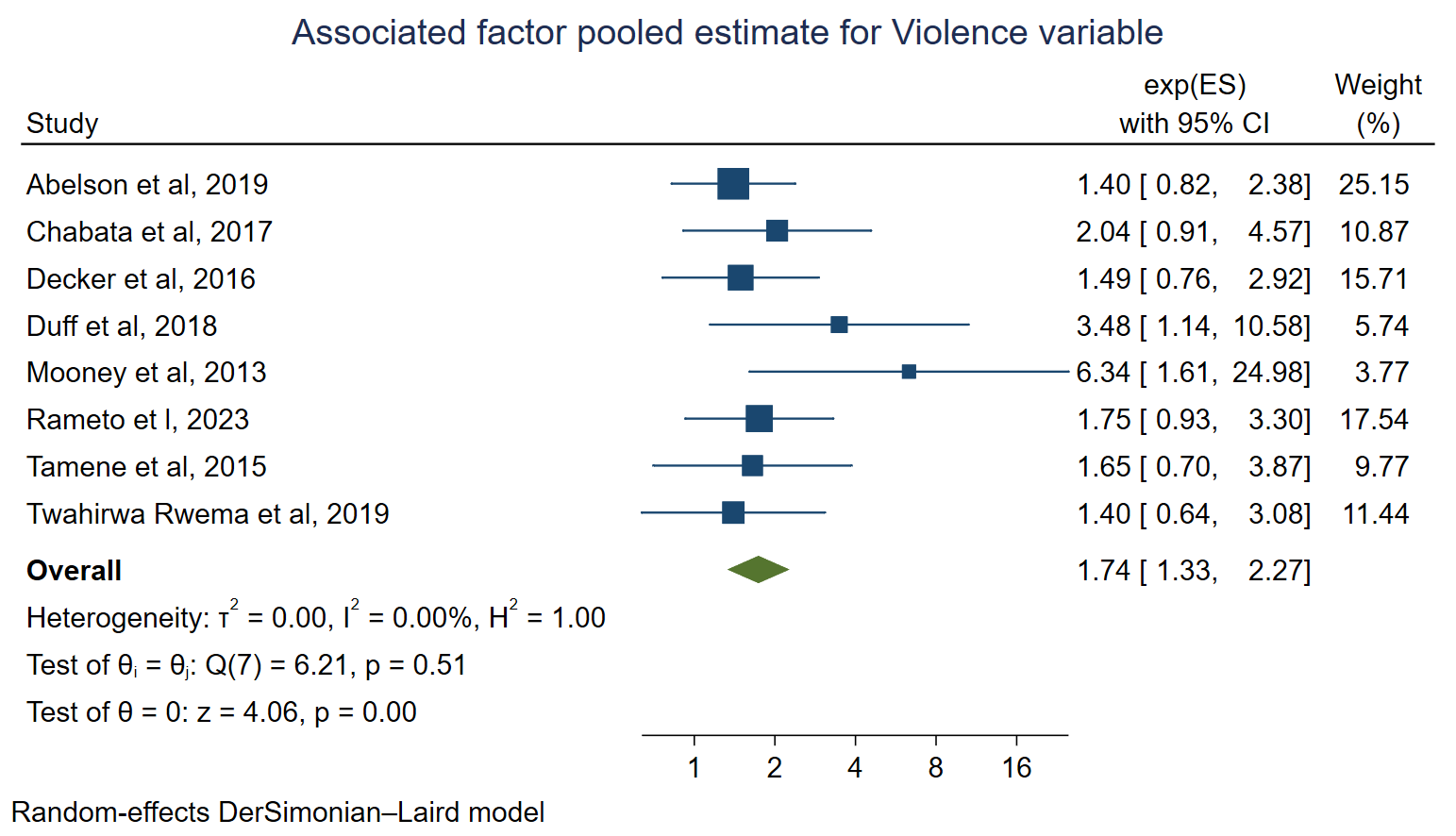


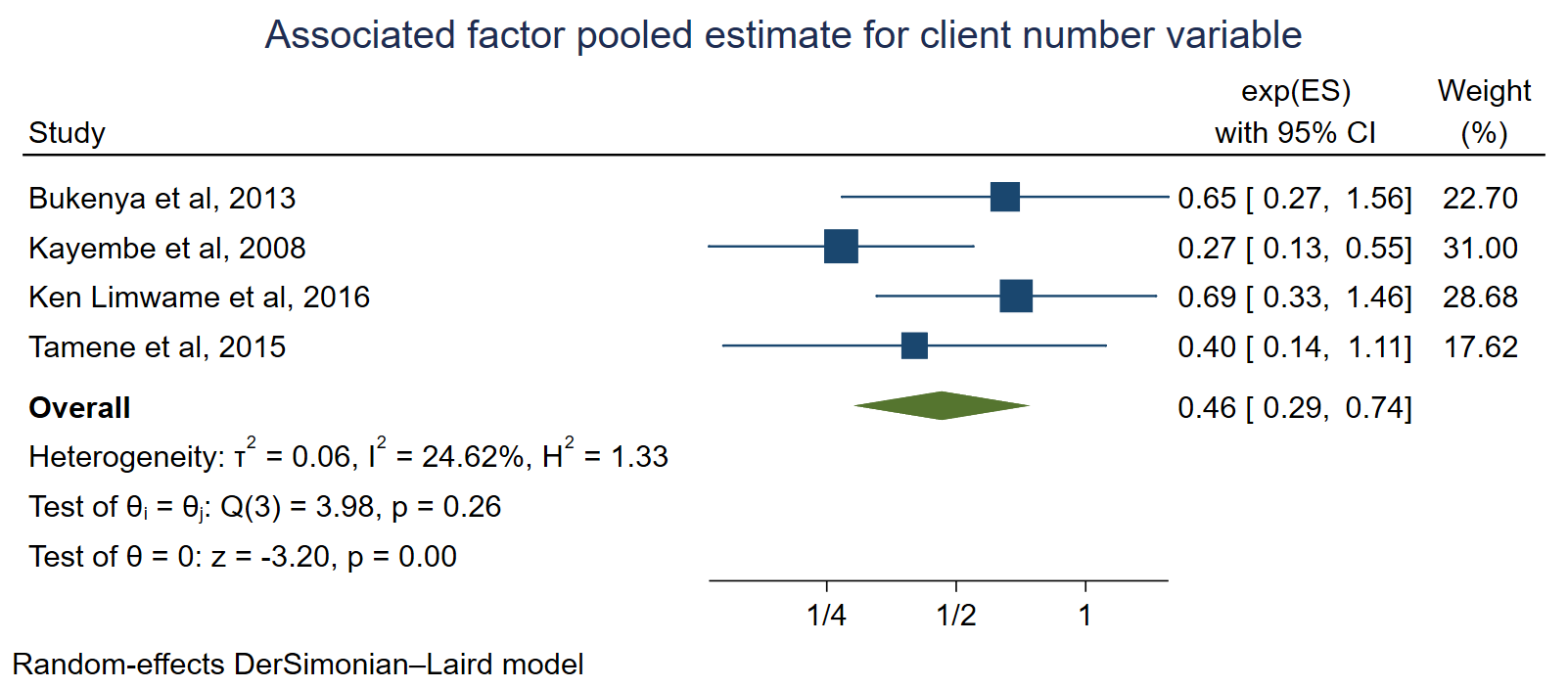

Supplement: S1 File — (DOCX) [file pone.0346903.s003.docx]
